# Supplementary material for: Proximity-effect-induced Superconducting Gap in Topological Surface States – A Point Contact Spectroscopy Study of NbSe2/Bi2Se3 Superconductor-Topological Insulator Heterostructures
Source: Sci Rep. 2017 Aug 9;7:7631. doi: 10.1038/s41598-017-07990-3 (PMC5550495; doi:10.1038/s41598-017-07990-3)
Supplement: Supplementary file 1 — Supplementary Information [file 41598_2017_7990_MOESM1_ESM.pdf]

Supplementary Information

Proximity-effect-induced Superconducting Gap in Topological Surface States – A Point Contact Spectroscopy Study of $NbSe_2/Bi_2Se_3$ Superconductor-Topological Insulator Heterostructures

Wenqing Dai, Anthony Richardella, Renzhong Du, Weiwei Zhao, Xin Liu, C.X. Liu, Song-Hsun Huang, Raman Sankar, Fangcheng Chou, Nitin Samarth, and Qi Li

This file includes

SI A. Point contact spectra of a $NbSe_2$/7 QL Bi_2Se_3 heterostructure

SI B. Point contact spectra of a $NbSe_2$/13 QL Bi_2Se_3 heterostructure

SI C. Magnetic field dependence of the proximity-induced Bi_2Se_3 bulk state gap

SI D. Additional gap-like feature at low temperatures in point contact spectra of a $NbSe_2$/13 QL Bi_2Se_3 heterostructure

SI A. Point contact spectra of a $NbSe_2$/7 QL Bi_2Se_3 heterostructure

We have measured point contact spectra of pure $NbSe_2$ single crystal and very thin Bi_2Se_3 films on $NbSe_2$. The pure $NbSe_2$ result was presented in supplementary information E of Ref. 1. Figure S1**a** plots the conductance spectra at different temperatures of a point contact on a $NbSe_2$/7 QL Bi_2Se_3 heterostructure and the fittings using the Blonder-Tinkham-Klapwijk (BTK) theory,[2] which is widely used to describe the transport between a normal metal and a superconductor with a finite transparency of the interface. A parameter Γ was included to describe the broadening effect.[3] The spectra were fitted well with the BTK theory and the energy gap value Δ and broadening parameter Γ from the fittings are plotted in Fig. S1**b**. Figure S1**c** shows the conductance spectra in different magnetic fields at 0.2 K and the Δ and Γ values from the fittings to the spectra are plotted in Fig. S1**d**. Here Γ is used, as a first approximation, to simulate the pair-breaking effect of a magnetic field.[3] The fitted energy gap Δ is ~ 1.0 meV at the lowest temperature, which is slightly lower than the pure $NbSe_2$ gap value ~ 1.26 meV from point contact measurement.[1] The gap value decreases with increasing temperature or magnetic field. The energy gap feature in the spectra disappears at ~ 7 K or under ~ 4 T magnetic field, which is consistent with the T_c and H_{c2} of $NbSe_2$ single crystals.

The measured superconducting energy gap in Fig. S1**b** and S1**d** are from the interface of the $NbSe_2$/Bi_2Se_3 which is dominated by the superconducting gap of $NbSe_2$ at the interface. This gap value decreases slightly from the bulk $NbSe_2$ gap and also decreases when the thickness of Bi_2Se_3 films is increased. These are consistent with the suppression of the energy gap in a superconductor at the interface with another material due to proximity effect.[4]

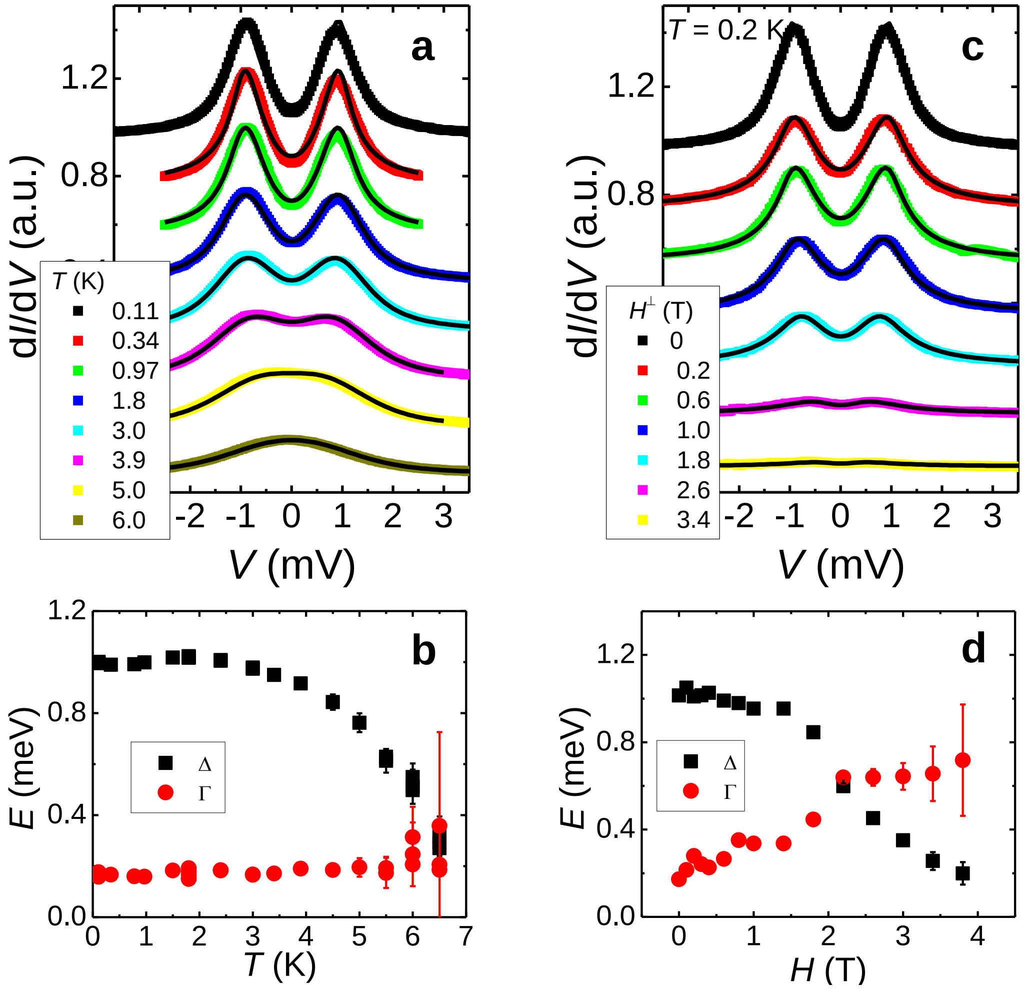

Figure S1. Point contact conductance spectra of a $NbSe_2$/7 QL Bi_2Se_3 heterostructure. **a,** Normalized conductance spectra of a point contact on the $NbSe_2$/7 QL Bi_2Se_3 sample at different temperatures. Curves are shifted vertically for clarity. The black lines are the BTK model fits to the experimental data. The contact normal resistance R_n is 485 Ω. **b,** The temperature dependence of the superconducting energy gap Δ and quasiparticle lifetime broadening parameter Γ values from the fittings in Fig. S1**a**. **c,** Normalized conductance spectra of the point contact on the $NbSe_2$/7 QL Bi_2Se_3 sample at different magnetic fields. The sample temperature is 0.2 K. Curves are shifted vertically for clarity. The black lines are the BTK model fits to the experimental data. **d,** The magnetic field dependence of Δ and Γ values from the fittings in Fig. S1**c**.

SI B. Point contact spectra of a $NbSe_2$/13 QL Bi_2Se_3 heterostructure

As we increase the Bi_2Se_3 film thickness, another conductance peak feature appears in the spectra and the spectra weight from the gap feature of $NbSe_2$ decreases. Figure S2**a** shows the conductance spectra of a point contact on a $NbSe_2$/13 QL Bi_2Se_3 heterostructure at different temperatures. At low temperatures, a conductance peak at low *V* bias ~ 0.3 mV appears besides the main $NbSe_2$ peak at ~ 1.0 mV. This gap feature is consistant with the proximity-induced bulk band energy gap. With increasing *T*, the two different peak features start to smear and become not resolvable above 3 K. Figure S2**b** shows the contact spectra at different magnetic fields at 0.1 K. The peak feature at ~ 0.3 mV is suppressed in small magnetic fields (see discussion in SI C). On the other hand, the $NbSe_2$ peak feature at ~ 1.0 mV remains unchanged in magnetic fields up to 20 mT. It disappears at ~ 4 T magnetic field, which is again consistent with the pure $NbSe_2$ H_{c2}.

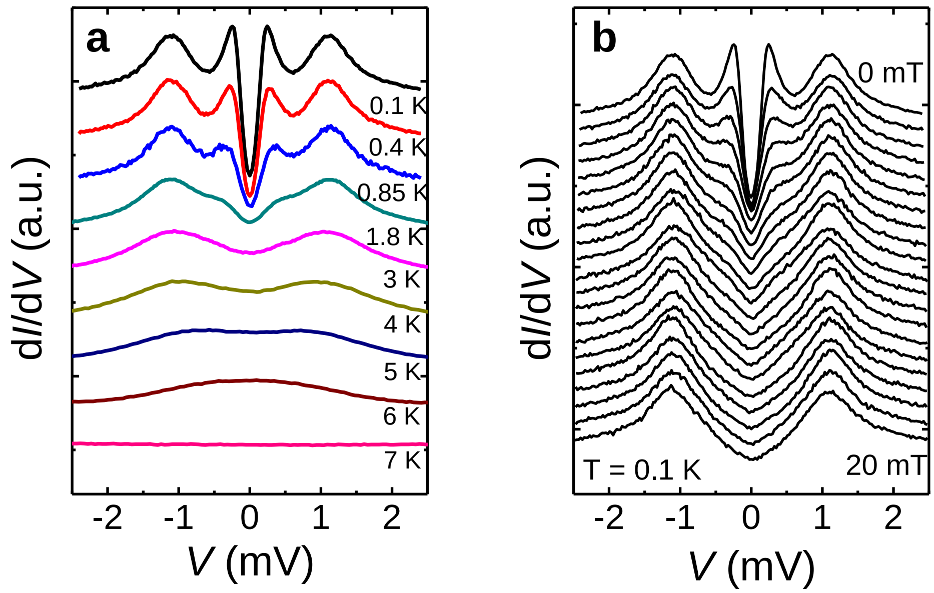

Figure S2. Point contact conductance spectra of a $NbSe_2$/13 QL Bi_2Se_3 heterostructure. a, Conductance spectra of a point contact junction on the $NbSe_2$/13 QL Bi_2Se_3 heterostructure at different temperatures. Curves are shifted vertically for clarity. **b,** The point contact spectra change with magnetic field from 0 to 20 mT at 0.1 K. Curves are shifted vertically. The contact normal resistance R_n is 26 Ω.

SI C. Magnetic field dependence of the proximity-induced bulk state gap in Bi_2Se_3

We studied the magnetic field dependence of the proximity-induced superconducting energy gap in the bulk of Bi_2Se_3. Figure S3**a** plots the point contact conductance spectra of the $NbSe_2$/16 QL Bi_2Se_3 heterostructure under different magnetic fields at 60 mK and the fittings using the BTK model. The energy gap Δ and broadening parameter Γ from the fittings are ploted in Fig. S3**b** together with magnetic field dependence data at 1.8 K. In PCS studies under magnetic field, Γ is often used to simulate the pair-breaking effect of a magnetic field in a first-order approximation.[3] We observed that the Γ is proportional to H at low magnetic fields. The Γ/H ratio is ~ 3 meV/T for both 60 mK and 1.8 K, giving further support to the validity of using Γ for field-induced broadening. The proximity-induced Bi_2Se_3 bulk state energy gap Δ decreases with increasing magnetic field. Although the conductance peak is nearly suppressed at H = 0.3 T, there is a finite energy gap Δ from the BTK model fitting. It is likely that the broadening from the magnetic field-induced pair-breaking smears the Bi_2Se_3 bulk gap feature in the conductance spectra.

It is worth noting that our point contact spectroscopy probes a surface area much larger than the vortex core. The PCS signal is from averaging a large area containing vorices cores and superconducting areas between vortices. The PCS does not have the spatial resolution to resolve a single vortex.

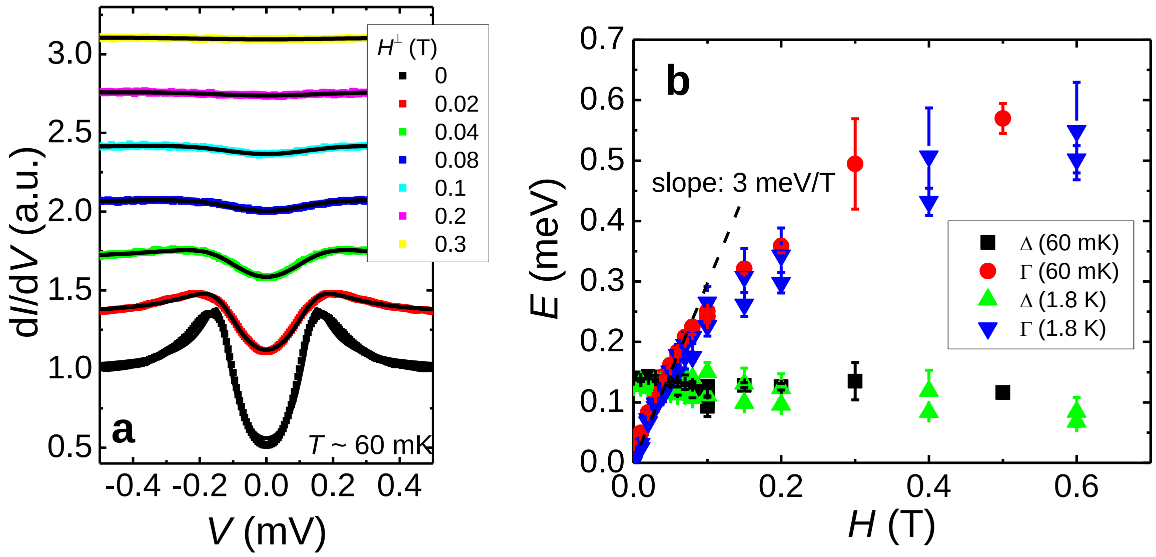

Figure S3. Magnetic field dependence of the proximity-induced gap in Bi_2Se_3. a, Pont contact conductance spectra of a $NbSe_2$/16 QL Bi_2Se_3 sample at 60 mK under different magnetic fields. Curves are shifted vertically for clarity. The black lines are BTK model fits to the experimental data. **b**, The magnetic field dependence of Δ and Γ values from BTK model fittings in Fig. S3**a** and fittings to point contact spectra at 1.8 K.

SI D. Additional gap-like feature at low temperatures in point contact spectra of a $NbSe_2$/13 QL Bi_2Se_3 heterostructure

We also observed the additional gap-like feature in a 13 QL Bi_2Se_3 on $NbSe_2$ sample at low temperatures. Figure S4**a** shows the point contact spectra at different temperatures on a $NbSe_2$/13 QL Bi_2Se_3 heterostructure and the corresponding BTK model fittings. Similar to the $NbSe_2$/16 QL Bi_2Se_3 sample, the spectra of this 13 QL sample do not have the $NbSe_2$ peak feature so the one-gap standard BTK model is used to fit the conductance curves. The standard BTK model does not fit the experiemental data well at low temperatures; The fitted gap value decreases at low T (circled in Fig. S4**a** inset). Following the same

method as used in the $NbSe_2$/16 QL Bi_2Se_3 sample analysis, a conductance cruve is calculated for T = 0.4 K and plotted together with the experimental conductance spectrum in Fig. S4**b**. the condutance difference between the experiemental curve and the simulated curve shows a peak feature at ~ 210 μV (Fig. S4**b** inset). The ratio of this second gap feature to the main gap is ~ 0.7, close to the two induced gap ratio in the $NbSe_2$/16 QL Bi_2Se_3 sample at 0.2 K, indicating that the second gap feature in 13 QL and 16 QL samples are are from the same origin.

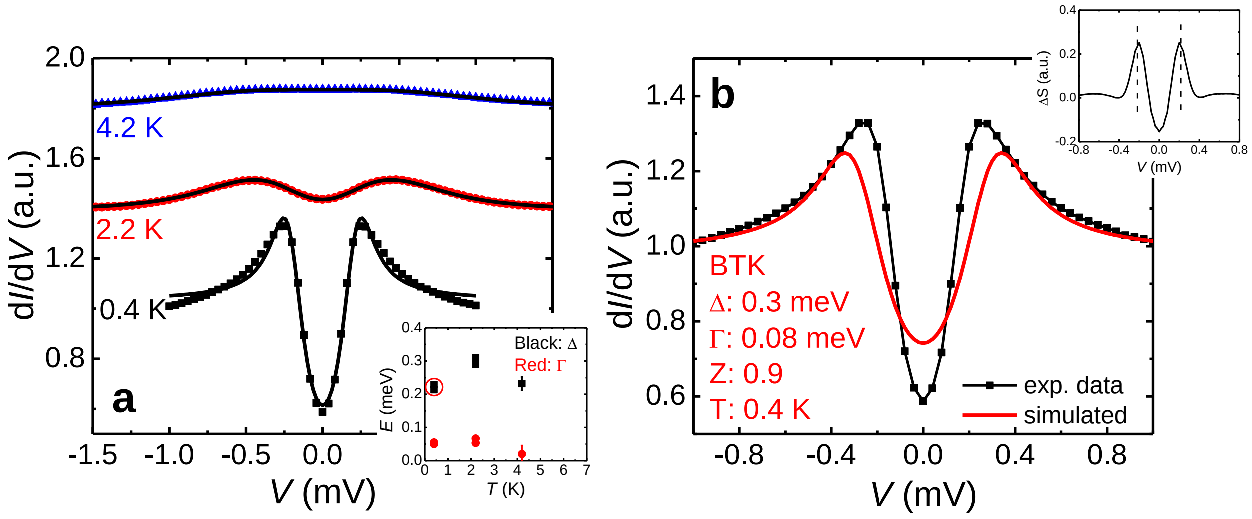

Figure S4. Additional gap-like feature in point contact spectra of a $NbSe_2$/13 QL Bi_2Se_3 heterostructure at low temperatures. a, Normalized point contact spectra of a $NbSe_2$/13 QL Bi_2Se_3 heterostructure at different temperatures. Curves are shifted vertically for clarity. The black lines are BTK model fits to the experimental data. The fitting parameters Δ and Γversus temperature are plotted in the bottom right inset. The contact normal resistance R_n is 22 Ω. **b,** The point contact spectrum at 0.4 K (black) plotted together with a BTK model simulated spectrum using the parameters shown in the bottom left of the figure (red). The top right inset shows the conductance difference between the experimental data and the simulation as a function of bias V. The dash lines mark positions of peaks at ~ 210 μV.

Reference

[1] Xu, S.-Y. *et al.* Momentum-space imaging of Cooper pairing in a half-Dirac-gas topological superconductor. *Nat. Phys.* **10,** 943–950 (2014).

[2] Blonder, G.E., Tinkham, M. & Klapwijk, T.M. Transition from metallic to tunneling regimes in superconducting microconstrictions: Excess current, charge imbalance, and supercurrent conversion. *Phys. Rev. B* **25,** 4515 (1982).

[3] Daghero, D. & Gonnelli, R.S. Probing multiband superconductivity by point-contact spectroscopy. *Supercond. Sci. Technol.* **23,** 43001 (2010).

[4] Gennes, P.G.D. Superconductivity of Metals and Alloys. (W.A. Benjamin, New York, 1966).
